# Values in University–Industry Collaborations: The Case of Academics Working at Universities of Technology

**DOI:** 10.1007/s11948-019-00144-w

**Published:** 2019-10-16

**Authors:** Rafaela Hillerbrand, Claudia Werker

**Affiliations:** 1 Institute for Technology Assessment and Systems Analysis (ITAS) and Institute for Philosophy, KIT - Karlsruhe Institute of Technology, Karlsruhe, Germany; 2 Department of Values, Technology and Innovation - TPM - Faculty of Technology, Policy and Management, Delft University of Technology, Delft, The Netherlands

**Keywords:** Values, Value conflicts, University–industry collaborations, Academics, Universities of technology

## Abstract

In the applied sciences and in engineering there is often a significant overlap between work at universities and in industry. For the individual scholar, this may lead to serious conflicts when working on joint university–industry projects. Differences in goals, such as the university’s aim to disseminate knowledge while industry aims to appropriate knowledge, might lead to complicated situations and conflicts of interest. The detailed cases of two electrical engineers and two architects working at two different universities of technology illustrate the kinds of problems individual scholars face in university–business collaborations. These cases are based on qualitative interviews and additional data and demonstrate that, while value conflicts emerge on the organizational level, it is primarily the individual researcher who must deal with such conflicts. This analysis adds to existing studies in two ways: first, it explicitly addresses normative issues framed in terms of ethical and social values, thereby going beyond the common social-science perspective of university–business collaboration. Secondly, it provides qualitative insights, thereby identifying details and issues not apparent in quantitative studies. In particular, it is evident that university–industry collaborations are prone to value conflicts not only in research but also in education and job training.

## Introduction

Western industrialized economies rely heavily on advances of science and technology. They are seen as vital in addressing the currently pressing societal goals, the so-called “grand challenges” of an ageing society facing increased environmental degradation such as climate change (e.g., European Commission 2013). Moreover, because of global competition Western industrialized economies rely heavily on generating knowledge and carrying out research and development in order to maintain economic welfare. As a consequence, integrating scientific research and technological advances has become increasingly important in recent decades. Policy makers, for example, want academic researchers to contribute much more to applied research, to technological development, to production and process development, and even to the dissemination of technology (Fromhold-Eisebith and Werker 2013; Martin 2012). Over the last decades, the desire to integrate scientific research and technological development has increased the importance of, and has changed the nature of, collaborations between universities and industry (Carayol 2003). Existing intermediary agencies do not suffice to integrate science and technology because their involvement extends the knowledge chain and leads to problems in knowledge creation and transfer (Etzkowitz and Viale 2010; Guerrero et al. 2012; Philpott et al. 2011). Therefore, a new form of university known as the “entrepreneurial university” has emerged as “… a gradual reinforcement of the ‘model of innovation centred on the university’. … This university gives birth to the dual academic career. Beyond the traditional truth seeking scientist, there is another scientist: the ‘entrepreneurial scientist’ who is able to interface knowledge and innovation” (Etzkowitz and Viale 2010).

As a result, university–industry collaborations are on the rise and research money spent at universities increasingly comes from industry.^1^ Governmental funding bodies encourage this trend. For example, with the current research funding schemes, “Corporative responsible innovation” (MVI—*Maatschappelijk verantwoord innoveren*), the national Dutch funding agency (NWO—*Nederlandse Wetenschappelijke Organisatie*) aims at starting and extending university–industry collaboration.^2^ Here only parts of the research are publicly funded; the participating companies have to provide substantial funding as well. Likewise, the German ministry of research and education (BMBF—*Bundesministerium für Bildung und Forschung*) imitated co-funding with the so-called “Leading-Edge Cluster (*Spitzencluster*) Competition” for more innovation and growth.^3^ The UK government even commissioned a report to review business-university collaborations in the UK to improve incentive schemes in order to improve university–business collaboration.^4^

Close collaborations between academia and industry may lead to possible conflicts. The goals of business and universities differ considerably: while industry is mainly profit-oriented, the primary goals of the universities are the production of knowledge (i.e., research results that are publicly accessible and usable) and its dissemination, particularly in the form of education (Martin 2012). These differences in goals as well as differences in funding sources may lead to ethical concerns. Basic research is generally funded with public money in the form of taxes or philanthropic donations distributed by government agencies or charitable foundations, respectively. The assumption upon which the funding is based is that this research will serve the public good. When the fruits of research are directed mainly to the private sector and the production of products that then make a profit and thereby benefit a relative few, ethical concerns arise. The literature addressing such concerns focuses on the implications that close collaborations between university and business have for research. In this regard, the life sciences, including biotechnology and health care services, have received much attention (e.g., Blumenthal et al. 1996a, b).

While academics do not seem to face negative changes in their academic output when collaborating with industry partners, they nonetheless hesitate to engage in university–business collaborations. Quantitative studies indicate that there is no obvious correlation between industry engagement and research performance in terms of journal papers published by researchers (Gulbrandsen and Smeby 2005). While this indicates that academic performance usually measured by publications is not affected by collaboration with industry, researchers at universities remain skeptical about university–business collaboration (Lee 1996). At the core of this skepticism lies inadequate treatment of the various conflicts faced by individual university researchers engaged in close business collaboration. Existing contracts and arrangements, for example about sharing intellectual property rights (Carayol 2003), do not cover all aspects of collaboration with industry, thereby leaving researchers to deal with them on their own. As a consequence, two-thirds of the nanotechnology researchers responding to a survey carried out by Katherine McComas (2012) worried that the existing rules were insufficient to guard the integrity of their research.

In order to identify and analyse value conflicts, the present study considers both the entrepreneurial scientist who is central to the integration of science and technology, and universities and industries. This study examines conflicts that emerge from the very different aims of academia and industry. In so doing, it explores the abstract values held by organizations and the impact that potential conflicts in values between their different employers’ goals have on individual researchers. This study does not consider other kinds of conflicts researchers face, such as conflicts of interest due to an individual’s interest in money or fame that may conflict with organizational values. In order to get empirical insights into these value conflicts, this study analyzes qualitative interviews with four entrepreneurial scientists from universities of technology where there is often a significant overlap between academic research activities, technological development and industry applications in engineering and architecture. This analysis complements existing research in the field in two ways: first, from a theoretical point of view it explicitly addresses normative issues within a value approach. Thereby, it goes beyond the common social-science perspective on university–business collaboration. Secondly, from an empirical point of view, it provides qualitative insights, identifying details and issues about value conflicts not visible in quantitative studies (Gulbrandsen and Smeby 2005; Lee 1996; McComas 2012).

## Theory: A Values Approach to University–Industry Collaborations

### A Normative Perspective on University–Industry Collaboration

As a starting point, the fundamental assumption of the study is that universities and industries as organizations aim at different goals. The conflicts that individual researchers who engage in university–industry collaboration face arise from these. Further, it is assumed that the perceived severity of the conflicts reported in the following sections hint at underlying unresolved ethical dilemmas. Consequently conflicts are treated not as mere conflicts of interest, but as ethical conflicts, in particular as value conflicts. As a result, the focus is on two levels: one, at the organizational level, that is, conflicting values of industry and university; and the second on how these conflicts are perceived and treated by the individual researchers. Additional conflicts, such as the individual researchers’ goal of increasing his or her income or academic productivity, are potentially related but go beyond the scope of this paper.

While general moral and social values are shared by industrial and university partners alike, individual researchers who bridge both kinds of organizations often nonetheless feel that they cannot solve value conflicts in a way that does justice to them both. Often academia and industry face not only contradictory goals, such as maximizing profits or publishing as much as possible in high impact journals, but also vague goals, such as solving the grand challenges of society, or transferring and applying knowledge from university to industry. Therefore, individual researchers in university–industry collaborations face vague goals on top of contradictory goals at the organizational level. As a result, it can be extremely difficult for the individual researcher to locate and handle value conflicts adequately.

Addressing the problems of individual researchers within university–industry collaborations in terms of value makes this theoretical framework part of applied ethics. Focusing on universities of technology, our ethical analysis may be located somewhere between the ethics of technology or engineering, and business ethics. Since the mid-twentieth century, there has been much research on the ethics of engineering and technology. While the latter considers the normative legitimacy of various technologies, the former examines engineers’ obligations to society, clients, and the profession (Vermaas et al. 2014). In addition to ethical problems arising from technical practice, considerations of business conduct have increasingly become important. For example, the American Society for Civil Engineers (ASCE) mentions legal compliance and relationships with clients, consultants, competitors, and contractors as central to the ethics of engineering (American Society of Civil Engineers 2000). As John Hooker puts it: “Engineers must now think about ethical issues that were once the province of business managers” (Hooker 2000). Analyses of organizational arrangements that may or may not facilitate ethical behavior (e.g., enabling whistle blowing) prove to be more and more necessary. Hence, in this field, there is a fair amount of overlap between the ethics of engineering and technology on the one hand, and business or corporate ethics on the other (Birch and Fielder 1994). However, the specific conflicts entrepreneurial scientists face have not yet received much attention in the normative literature.

Nevertheless, the interplay between industry partners and academic researchers working at universities or publicly funded research organizations is of central relevance for applied ethical reasoning. In particular, including publicly funded research in joint research with privately owned firms is frowned on because universities are mainly financed by public money. When using labs or manpower in university–industry collaborations, some public money automatically benefits the specific private companies involved in the collaboration. At the same time, universities aim to generate and disseminate knowledge that can be applied to societal challenges. To achieve this, the universities must tailor knowledge to potential users, ranging from policy makers to industrial partners. Particularly for universities of technology, this means engaging in university–business collaborations. To govern these kinds of situations there are rules in place, for example:

We strive to remain current in respect to the dilemmas and the social dimensions of work in our field. We further strive to avoid potential conflicts of interest, via transparency about our methods, intentions and results, and we are called upon to bring any possible conflicts to the attention of the university. Integrity and open inquiry are essential for the workings and reputation of science, and as researchers at TU Delft we are expected to act accordingly (Delft University of Technology 2019).

In formulating these rules, Delft University of Technology allows individual researchers significant latitude in their activities when collaborating with industry. Of course, such freedom reflects the dilemma faced by universities of technology: on the one hand, publicly-funded universities should produce goods that are publicly available, and on the other hand, they can only contribute to advancements in technology and innovation by sharing knowledge in collaboration with partners from industry, who want to privately appropriate at least parts of the outcome. This is not only problematic in cases where the industrial partner explicitly requires that the publicly-funded knowledge be considered proprietary and kept secret, but also in cases where academics work on industrial projects as they provide cheaper input to the knowledge production for (some parts of) industry. Their labor is not available to pursue research goals that are set by universities and thus, in these cases the intention of publicly-funded research may not be attained. University–industry collaborations lead to complex and complicated conflicts of interest and only broad rules like those cited above assure that knowledge sharing with collaboration partners that is essential for achieving important societal goals, is not hampered (Delft University of Technology 2019). At the same time, the broad scope of the rules means that university researchers themselves must evaluate each situation in which they engage with industry in light of potential conflicts and discuss them with peers and other stakeholders. This additional uncertainty and effort can impede collaborations with industry.

### Values in Applied Engineering Ethics

Contemporary ethical theory and, to a large extent, applied ethics, often take action(s) or agency as a starting point (Mitcham 2005; van de Poel and Royakkers 2011). Classical modern ethical theories are concerned about what makes an action right or wrong, either in terms of its outcomes as in consequentialism, or in terms of intentions or obligations as in deontology. The recent revival of virtue ethics in the twentieth century which also left its traces in ethics of engineering and technology (e.g., Harris 2008) does not evaluate individual actions, but rather focuses on individuals and their intentions.^5^

In consequentialist, deontological, or virtue ethics, it is the individual who is center-stage, either directly in terms of being an agent, or in terms of her actions. Here, we hold that at the heart of the broader agenda of analyzing university–industry collaboration from a normative perspective lies the need for analyzing university–industry collaboration in terms of values. This approach has been argued for other areas of applied ethics as well, particularly engineering ethics and the ethics of specific technologies (e.g., Johnstone 2007). Using a value-based approach makes it possible to go beyond the limitations of evaluations in terms of actions or agency so that the complexity of university–business collaborations can be considered. Often indirect (side) effects of development and collaboration processes are difficult to estimate and impossible to trace back to individual agents. Moreover, a normative assessment in terms of agency does not adequately reflect the reality of individual researchers who often cannot predict the outcomes of their actions, nor the wider societal implications of those actions. Given the context of university–industry collaborations with its complex interactions between individuals who are aiming at innovations which are not fully predictable or controllable, an ethical evaluation in terms of agency seems untenable. Though research teams may be able to anticipate and take more risks than individual researchers, it is unrealistic to expect to capture all possible ethical issues in this way. Within applied ethics of technology and engineering, large parts of the discussion center on the inability to assign responsibility to individual agents. Even where individual agents can be identified, as is often the case in university–industry collaboration, e.g., in the context of information and communications technology (ICT) research

…non-linearity, opaqueness, positive feedback loops and complexity mean that agents are frequently unable to predict the outcomes of actions, assess the potential for unintended negative consequences, or even clearly distinguish causes and effects (Johnstone 2007).

Development of new production processes or products (e.g., in the form of prototypes), is often a complex process, with uncertain results not only regarding the product but also regarding the process of research. University–industry collaboration takes place off the beaten path of industrial production. It is about entering unknown territory or at least about gauging the unknown. Collaborations are one way of dealing with the inherent uncertainty that comes with research at the technological frontier (Etzkowitz and Leydesdorff 2000). At the same time, inherent uncertainty leads to even more complexity in deciding on ethical issues in university–industry collaborations, in particular because the nature of the relationship changes over time.

Analysing university–industry collaborations in terms of value is of practical value as reliability or efficiency can be seen as technological or economic values that are used in design. For example, the ways engineers phrase design requirements can often be straightforwardly reconstructed in terms of values, despite the fact that they do not explicitly use the term “value”. Moreover, firms as well as universities often articulate core values in their mission statements (van de Poel and Royakkers 2011). The mission statements of the universities of technology at which the four interview partners work are good examples. In its mission statement Delft University of Technology refers to values such as sustainability and safety (Delft University of Technology 2019); RWTH Aachen explicitly states the “Values at the RWTH” (RWTH Aachen University 2013) that aim at conducting interactions with respect to teaching and research both within the university and with external partners. While the mission statements demonstrate the desire of the university management to reflect ethical issues and values, it is not clear whether they also express the point of view of individual researchers and whether they guide their actions.

Values quite generally refer to what is worth striving for and may be distinguished from preferences of the individual researcher. Values as the term is used here, are normative: the underlying claim is that people or organizations *should* strive for realization of these values. While individual preferences, for example the career perspective of the researcher who engages in university–industry collaboration, certainly plays a central role in how value conflicts emerge and are resolved, this paper focuses on the general values on which organizations build their activities. Thereby we distinguish between intrinsic and instrumental (also called extrinsic or functional) value (Zimmerman and Bradley 2019). Something of intrinsic value is considered as valuable in itself. For example, knowledge generation is one of the aims of a university and therefore valuable in itself from the perspective of a university. Likewise making profit is intrinsically valuable for business. In contrast, something of instrumental value is of value only because it helps to realize another value. For example, companies can profit from knowledge generation, though this is not their actual aim. Hence, for businesses knowledge generation is of instrumental value if it helps to increase the company’s profit (e.g., by leading to the design of a new product, or increasing the efficiency of existing processes).

## Methodology: Research Design and Data

### Basic Versus Applied Research at Universities of Technology

While the role of universities in general and universities of technology in particular have changed considerably (Fromhold-Eisebith and Werker 2013; Martin 2012) their research activities are often still classified by the categories of basic versus applied research stemming from the usage of Vannevar Bush (1945). In this approach, firms invest private money and produce private goods, aim at making a profit, and are entitled to privately appropriate it. In contrast, universities are financed by the general public and produce public goods, in particular publicly available knowledge (Balconi et al. 2010; Bush 1945). Hence, it stands to reason that the results of research at universities should be publicly available. From a policy point of view, the distinction between basic and applied research has served as a motivation to subsidize basic research at universities, thereby strengthening the role of academic researchers in basic research. The traditional distinction between basic and applied research (Bunge 1985; Bush 1945) seems today somewhat outdated and many have convincingly argued against it (Baird 2004; Balconi et al. 2010; Pitt 2000).

For universities of technology the notion of basic versus applied research has always been problematic. In particular, engineering sciences go well beyond a straightforward application of findings from the natural sciences (Balconi et al. 2010; Baird 2004; Pitt 2000; Stokes 1997). Moreover, in recent years academia has been increasingly requested to engage in applying results of research and to collaborate with industrial partners. Not only do policy makers ask for such involvement from academia (Fromhold-Eisebith and Werker 2013; Martin 2012), but universities of technology in particular also commit themselves to addressing societal goals with their research, for example in finding solutions to the grand challenges.

### Building the Empirical Basis with Stokes’ Quadrants

**Fig. 1:**
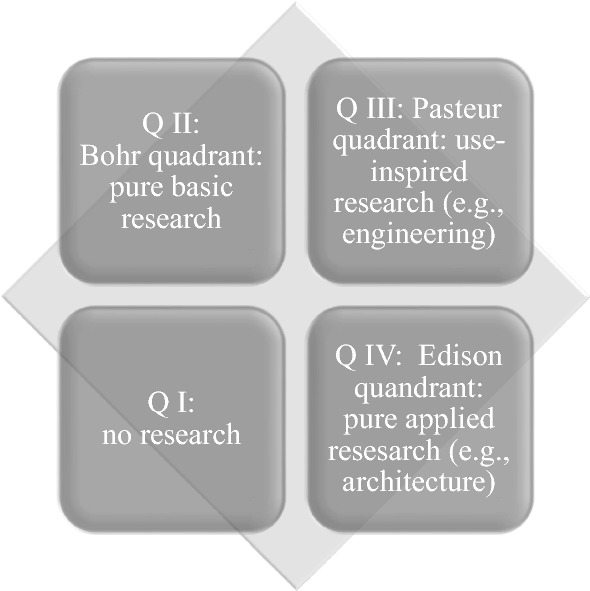
Basic and Applied Research Revised, inspired by Stokes (1997, p. 73): Vertical axis: quest for fundamental understanding; horizontal axis: consideration of use

To find interview partners at universities of technology who can clarify value conflicts emerging in collaborations with industrial partners the work described here draws on the well-known concept established by Donald E. Stokes (1997). This classification of types of research helps identify relevant interview partners as it captures the two major aspects of research activities. Stokes (1997) distinguished the quest for fundamental understanding from consideration of use. While the vertical axis in Fig. 1, the so-called Stokes-quadrant, indicates the degree to which research is done in pursuit of fundamental understanding, the horizontal axis indicates the extent to which research activities aim at solving specific practical problems of individuals or groups. The resulting four quadrants are Quadrant I (Q I), no research is taking place; while Quadrant II (Q II) represents pure basic research where the quest for fundamental understanding is primary. Quadrant IV (Q IV) represents pure applied research where development of practical, usable results is centre-stage. Quadrant III combines the quest for fundamental understanding with considerations of use. While this kind of research is often not captured within classifications of research and engineering, it is use-inspired basic research that contributes substantially to innovation and technological change in industrialized countries (Stokes 1997).

In this study, interviewees were selected based on Stokes’ concept. While generally speaking, the matched-pairs approach aims at using comparable data, we built a sample using theoretical constructs (Eisenhardt and Graebner 2007; Fromhold-Eisebith et al. 2014).^6^ Two scholars were selected at each of two universities: Delft University of Technology and RWTH Aachen University. At each university one member of the matched pair of interviewees was doing mainly *use*-*inspired basic research*, specifically in engineering. The second member of the pair at each university was engaged in mainly *pure applied research*, specifically in architecture. As a consequence, there are two sets of matches, both representing scholars doing a different type of research. The scholars in each set were matched regarding scientific discipline and seniority.

While in the Bohr quadrant of pure basic research collaboration of academics with industry are rather unlikely, they usually occur in the Pasteur-quadrant of use-inspired research and the Edison quadrant of applied research. Accordingly, we analyzed value conflicts for academics engaged in university and industry activities in the Edison quadrant and in the Pasteur quadrant. By using sets of scholars working in these quadrants, it is possible to tease out the existing and potential value conflicts. One set of scholars works mostly in the so-called Pasteur’s quadrant (i.e., engineering). Here, academics contribute to the fundamental understanding of their field and its potential application. The other set of scholars are architects who work mostly in the so-called Edison quadrant of purely applied research. The data consist of the contents of semi-structured interviews of about 1 h, and data derived from web-based research, in particular personal and professional webpages as well as publication databases. The first set of scholars consists of engineers working on microelectronics in a broad array of applications. The scholar at the RWTH Aachen University (RWTH E) has a distinct regional and international network of collaborators. This engineer has been closely cooperating with a number of small and medium-sized enterprises. His counterpart at Delft University of Technology is well connected on the regional, national and international level, with very close ties to Asia (TUD E). The second set of scholars contains two architects. One is combining his part-time position at Delft University of Technology with running his own architectural office (TUD A). His counterpart at RWTH Aachen University works as a researcher at the university and consults outside the university by using the legal and organizational framework of RWTH Aachen University (RWTH A).

### Interviewing Researchers at Universities of Technology About Value Conflicts

The data stem from interviews at RWTH Aachen University and Delft University of Technology carried out in 2011 and 2012. Both universities belong to the so-called IDEA League, a network of five leading European universities of technology and science (IDEA League 2019). This means that all our interview partners have similar backgrounds by being affiliated with universities of science and technology located in the European Research Area. At the same time, the interview partners differ because of their specific national background and because of their different disciplines. Universities of science and technology are commonly regarded as prime sources of knowledge to be transferred to industrial partners and as hubs around which new technology firms tend to cluster. The interviews emerged not only as a result of on-going discussion within the universities, but also because of an obvious need to clarify conflicts going hand-in-hand with researchers’ work. For example, at Delft University of Technology, discussions dealt with ethical conflicts arising in the research life and have been mirrored in a living document where the current state-of-the-art is reflected (Delft University of Technology 2019). At RWTH Aachen University, there were discussions on how to increase the number of patents (Hoffmann 2010).

While the analysis of value conflicts presented here relies on the framework outlined above, the interviewees were not informed of this framework, thereby adhering to frequently accepted guidelines for qualitative research (Eisenhardt and Graebner 2007). In selecting the qualitative approach chosen here it is essential to keep a particularly open mind to theoretical insights that emerge from the data. Consequently, this project began with a rather broadly defined research question (Siggelkow 2007). It is of particular importance not to formulate any hypotheses or relationships between the constructs in advance, thereby trying to avoid “[…] preordained theoretical perspectives or propositions (that) may bias and limit the findings” (Eisenhardt 1989, p. 536). In order to avoid biases due to the interviewer, the interviewer used neither leading questions nor any concepts discussed in this paper. In particular, neither the concepts of “value” nor “value conflicts” were introduced by the interviewer. In so doing, it was possible to avoid putting words in the mouth of the interviewees, while at the same time gathering information on the various tensions perceived by the researchers themselves. Interviews were conducted with four academic scholars asking questions about their research, their collaboration partners and their research output.

## Empirical Evidence: Value Conflicts in University–Industry Collaborations

For universities of technology as for any university, knowledge generation, knowledge dissemination and education are of inherent value, while for firms the inherent value lies with safeguarding return on investment as this is vital to the existence of the firm including employment for their staff. These values as well as several deriving from these might lead to conflicts. The value conflicts are examined for these four individuals (two engineers and two architects) as they might emerge in university–industry collaboration. The conflicts are grouped into five types: (1) students working in exchange for practical training, (2) public funding of applied research used to produce private profit, (3) outside personal earnings of publicly funded researchers, (4) universities not being compensated for industrial use of research, and (5) unfair competition resulting from the involvement of a publicly funded partner.

### Value Conflict 1: Students Working in Exchange for Practical Training

Universities of technology educate engineers and architects, i.e., professions with fairly clear-cut job profiles. In this respect, education in engineering and architecture more resemble education within the medical sciences, and are less like education in the humanities or sciences^7^: teaching does not only consist of a rather narrow academic focus on knowledge dissemination, that is, lecturing in a narrow sense, but also training. Just as future physicists in their academic training must learn not only about existing theories and knowledge in the field, but also about the “lab”-practice of how to *do* research, future engineers need to learn how to advance their field. As this is regularly done outside the academic realm and within companies, education at universities of technology often encompasses training on the job as well as pure knowledge dissemination through university lectures. Indeed, certain aspects of engineering practice can only be learned by taking part in that practice, e.g., internships with industry are necessary for a full engineering education. Students benefit immensely from being taught in the context of real problems. Hence there is significant overlap between the aims of universities of technology as regards this aspect of education, and the interests of firms in good, qualified (student) workers. In the case of student workers labor is also cheap, forming an additional incentive for industry to collaborate with universities. Despite these mutual interests differences in values might lead to problems, e.g., (1) while students carry out work for the firm might help the firm, it may not benefit the students’ education because it is not sufficiently related to it; (2) students have to work too many hours; or (3) they might not be adequately paid by the firm.

**Fig. 2:**
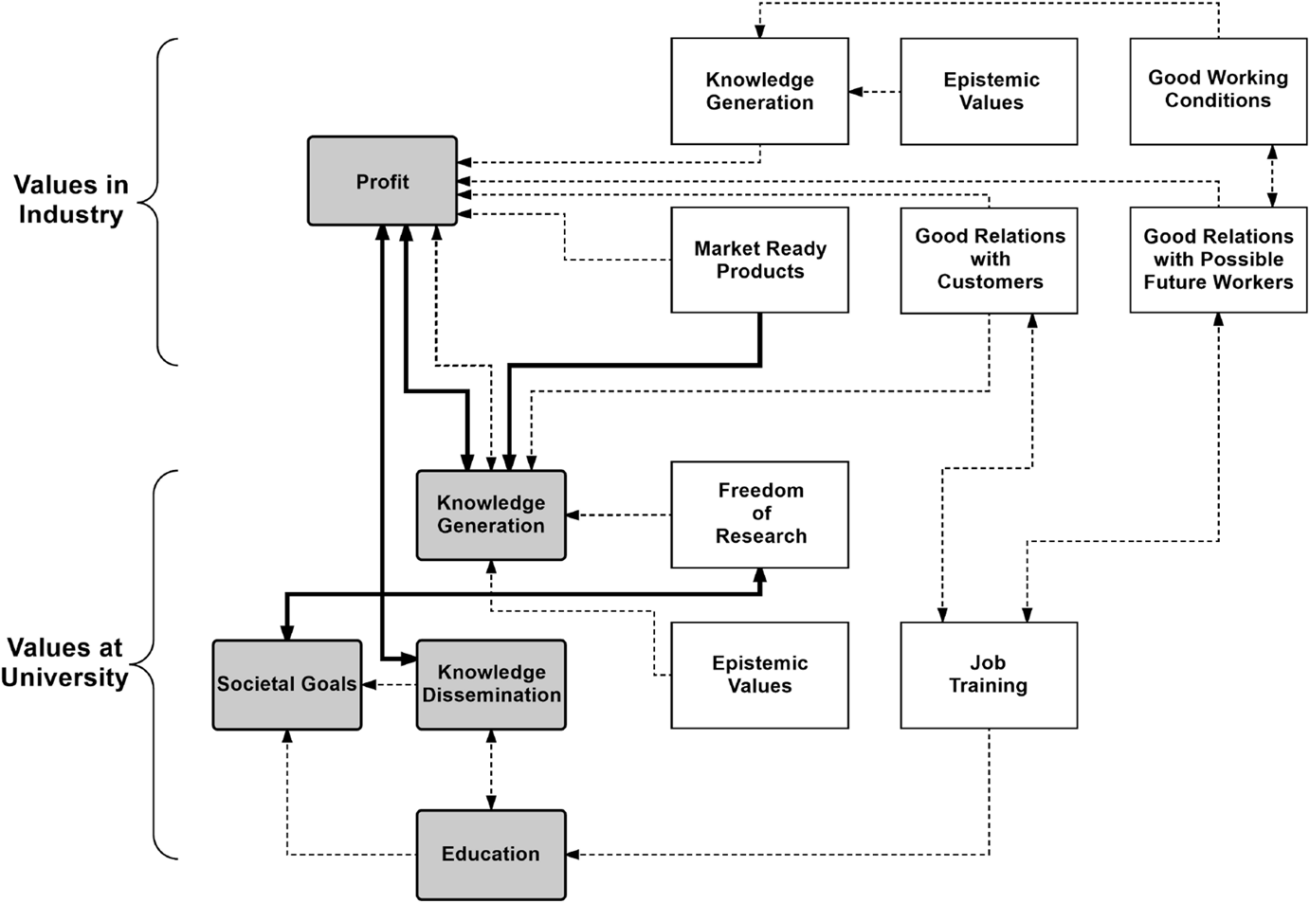
Typical value conflicts of industry (upper) and universities (lower) are displayed. Intrinsic values are on the left (shaded blocks); instrumental values are on the right (white blocks) (see also Table 1). Typical conflicts between values are indicated with a solid line and arrow, while a dashed line indicates an instrumental relationship where the implementation of different values are mutually reinforcing

The architect working at RWTH Aachen University (RWTH A) explained that his students profit from his relationships with the private sector, because they get the opportunity to learn from real world problems. For example, the architect contributes to the land development plan of a city. While the majority of the work is done by a privately owned architect’s office, students attending seminars that relate to the work of RWTH A are not asked to do any direct chores related to it but learn about the whole process. According to RWTH A, the advantage of these kinds of seminars is that students learn not only about successfully finished projects, but are involved in the project process itself. (RWTH A 00:08:43-9 to 00:09:26-8). This is a good example of implementing a mutually beneficial arrangement (see Fig. 2): the university benefits because it can improve the training of its students. At the same time, the industry partner obtains knowledge that is current state-of-the-art in research as well as contact with good students who might be interested in future employment. Most of the time, this kind of collaboration between universities and industry is initiated by university researchers in order to improve student’s training.

While the actual case does not show any value conflicts, it has the potential for a severe conflict. If, contrary to the case at hand, industry does not see sufficient reason to get involved in teaching as the balance between various instrumental values is set differently, it becomes tempting to offer students work in exchange for their opportunity to learn from real cases. In this kind of situation, students might be exploited because the work that they do in exchange for obtaining experience in the private sector might be worth much more than it costs the private sector to train them. It would be the task of the university to assure a fair solution considering both the need for firms to receive some compensation for their contribution to teaching, and for the students not to be exploited as cheap labor. So far, finding such a fair solution is up to researchers who teach the students. It would help the researcher if the university would facilitate this by developing appropriate regulations available to the individual researcher.

### Value Conflict 2: Public Money Spent on Applied Research to Make Private Profit

Unlike the sciences or humanities, engineering and architecture are committed to real world practice. In particular, engineers and architects apply their knowledge in order to design, build or maintain artifacts in the broadest sense, ranging from machines or other devices to structures or processes. Value conflicts of university–industry collaboration may lead to situations where the outcome of the development of prototypes is inherently uncertain. It might be that firms are reluctant to invest in this kind of prototype because it goes hand-in-hand with the risk that no market launch is possible. At the same time, the development of the prototype might be of instrumental value to universities because it may provide a first step to solving real-world problems. The obvious conflict here is that the firm makes money with the market launch of the product, while a lot of research money that went into the design of the prototype was done by university researchers and thus was financed by the general public. In specific contexts, such conflicts may be partly resolved by drawing a clear line between knowledge generation and prototype generation on the one hand, and large-scale production on the other hand. As the engineer working at RWTH Aachen puts it regarding the firms with which he collaborates:

Of course we stay in contact with them, but we … only cooperate in the development of the product up to [a] certain point where we as a university say we are not [a] manufacturing industry, we can only produce a limited number of the thing. We cannot give any guarantees and cannot give a CE^8^ label … and then we are characterized by education. … then we need to say okay, guys, you can do this for a licensing fee or whatever, then this dissolves. … then they are just gone. And say, okay, it was a good time but you have nothing to offer us at this time. (RWTH E, 00:35:07-7, translation).

Whether or not such a clear cut line between prototype generation and large-scale production can be drawn can only be decided on a case-by-case basis. Here it is important to ask whether the university–industry collaboration contributes to general knowledge within the respective discipline as a whole, and not simply to creation of a prototype as the precursor to mass-production (cp. RWTH E, 00:32:51-9).

Value Conflict 1, emerging from the need to involve industry in the training of engineers-to-be, and Value Conflict 2 have obvious and significant overlap. Consider as a generic example, the conflicting interests that emerge from the knowledge dissemination and implementation associated with spinning off companies. The arising conflicts for the university are clearly expressed by the engineer working at Delft University of Technology:

We have a student …, he starts up a company. So a few companies have been started up here. So that’s important. I, myself, am too busy to make a link between what we are doing here, because what we are doing here is always small scale and prototyping for the big industry. So it is important, but there’s always a conflict between your scientific output and your personal interest in that company. Sometimes technicians have a feeling they are there to make the professor rich and not the university. So, it is important, but I’m not always so happy about the way it goes (TUD E 01:04:40-5).

Spin-offs are a widely desired output of universities of technology, although they do not often emerge in a direct way and might come with problems regarding value conflicts. While the architect working at Delft University of Technology has no direct spin-offs out of his group, he knows that many of his former students start their own businesses:

**TUD A**: Spin-offs in an indirect sense of course we have. There are offices: young graduates, they’ll start an office and do their profession. And they will not do it with us, but they do it[?] with the knowledge that we gave them. But this is not…

**Interviewer**: It’s not like they developed an idea here and they start an office with that…?

**TUD A**: No, not that I’m aware of (TUD A 00:54:02-6 to 00:54:09-8).

Though TUD A is not aware of actual problems, there is the potential for severe conflicts. Suppose the students use knowledge developed in the university in their start-up. Despite the university’s aim to spread knowledge, the question arises as to whether there is money to be made from that particular knowledge, should not the university have its share. Then industry could indirectly pay back the taxpayer who initially funded the university research that made the spin-off possible. As always, “the devil is in the details” and a discussion as to where lies the boundary between acceptable and unacceptable behavior is beyond the scope of this paper. The following citation from the interview with an engineer at RWTH Aachen University illustrates this as it shows how much the intricate relationship between industrial and university partners changes over time. The relationships with a spin-off from Helmholtz Institute, a scientific-technical research center with 70% of its annual budget raised from public funds, is described by RWTH E:

And we collaborated with them for years, more than ten years, and then we could not collaborate with them anymore, because they were going too much into the production, and then they could not stay in close contact with a university institute anymore (RWTH E, 00:32:51-9, translation).

### Value Conflict 3: Personal Earnings of Publicly-Funded Researchers

Value Conflict 3 identifies a problem that might occur in every university–industry collaboration when the university researcher is compensated for her efforts in solving industrial problems. While there are rules in place guiding researchers in this kind of situation (RWTH Aachen University 2013; Delft University of Technology 2019), it is always difficult to determine when a publicly funded researcher relies on either resources or time for which she is already compensated when working for the industrial partner. While RWTH E does not receive money from the company after the prototype is developed (see above), suppose he had been reimbursed for his efforts in helping to develop the prototype. Then, the question would arise whether he could privately accept the money. Because he is paid with public money while consulting on production of the prototype, it stands to reason that he should reinvest the money in research activities at the university.

At universities of technology it is fairly usual for senior university researchers to combine their position at the university with having their own company, e.g., TUD A, the architect who works at Delft University of Technology part-time and also has his own architectural office. TUD A did not recognize any conflicts in this context and yet there are strict regulations as to under which circumstances researchers may work in addition to their university position. Further, complications might emerge if the researcher’s students do work for the researcher’s company because this may improve the profit of the company. Hence, through their university affiliation researchers may experience personal financial benefit for their own companies.

### Value Conflict 4: Universities are Not Compensated for Industrial Use of their Research

While the above discussion raises concerns about academics receiving personal income from work they do as public servants, at the same time, receiving no compensation from industry for commissioned work is also problematic. This point is illustrated by both architects. The one working at Delft University of Technology addressed the problem like this:

**Interviewer**: Would you say that you have a lot of customers/clients in the region of Delft?

**TUD A**: Well. Yes. We want to have these clients [laughing], but it’s hard to get them to pay (TUD A 00:04:14-2 to 00:04:23-9).

His counterpart at the RWTH Aachen University has similar experiences:

**Interviewer**: Could one summarize that in cases of collaborations with local councils they almost always approach you?

**RWTH A**: Well, it has to be formalized first. If there is a kind of assignment there has to be [a] call for proposals first. Usually, there have been professional contacts with these people in the past. If someone approaches me who does not know me, it often happens that in 80% to 90% we simply cannot do it. People sometimes have rather strange ideas about what one can quickly produce for them which does not work like that, if you tell them that you need money for the chair^9^ and have to employ people, then they quickly say that they did not imagine it like that. There are relatively many blind requests that come to nothing. Everything else where I know what they do and they know under which conditions we contribute, is naturally much more stable (RWTH A 01:10:02-9, translation).

He points out that when cooperating with private offices he has to give 40% overhead costs to the university. This means that many of the small consultancy projects are not possible. Because he values the inspiration he receives from these projects, he continues to cultivate these relationships (RWTH A 01:10:02-9).

### Value Conflict 5: Unfair Competition Resulting from Involvement of a Publicly Funded Partner

Not only for universities but also for companies collaboration might lead to value conflicts. This holds particularly for competitors of firms collaborating with universities of technology. The quotation from RWTH A above illustrates very clearly that collaborations with local councils do not work because of worries about unfair competition. While the university researcher already has his office and some staff are publicly funded as a result of his function as department chair at the university, privately funded competitors would have to bill the full costs of their working hours. At the same time, in order to advance knowledge of his field, RWTH A relied on these contacts outside the university. He was worried that his work would dry up without these collaborations and was looking for new forms of collaboration with local councils and private offices.

## Towards a Values Approach to University–Industry Collaborations

Ethical values like health, welfare, or justice are often reflected in corporate or engineering values in the form of honesty (e.g., as a principle that guides communication in a firm), safety of launched products or working conditions, quality, efficiency, and so forth (cp. Kaptein 2004). Though it seems safe to assume that underlying moral values and many social values are shared by firms as well as universities, the two differ significantly as to what they hold valuable per se, i.e., they have different intrinsic values. Intrinsic values of a firm generally depend on their owner structure and commonly include guaranteeing the survival of the firm and earning returns on investment for the owners. In Table 1, the former are subsumed under “values articulated in the mission statement” (B), while the latter is referred to as “profit” (A). Concern for good working conditions (a), launch of a new product (e), or research for developing a prototype (f) have an instrumental value for firms. These and other instrumental as well as intrinsic values that are held by industry, and how they relate to each other, are depicted in Table 1. Note also that intrinsic values may be instrumental for other intrinsic values. For example, the values formulated in the mission statement of a private business may indirectly contribute to profit. Table 1 lists potential mutual reinforcement of different values: values reinforcing other values are indicated in parentheses.

**Table 1:** Intrinsic and instrumental values of industry and universities of technology

	Industry	University of Technology
Intrinsic values	(A) Profit (B) Values articulated in mission statement (A, 1)	1. **Knowledge generation** (4) 2. Knowledge dissemination (4) 3. **Education** (1) 4. Societal goals
Instrumental values	(a) Good working conditions (A) (b) Good workers (A) (c) Good relation to costumers (A) (d) Good relation to possible future workers (b) (e) Market-ready product (B) (f) *Proto*-*type* (e) (g) **Knowledge generation** (e,f) (h) Various epistemic values (g,e,f) (i) *Job training* (d) (j) **Education** (i, g) (k) Safeguarding employment (a) …	(i) *Job training* (3) (ii) Epistemic values (1) (iii) *Prototype* (2) (iv) Freedom of research (1) …

The numbers and letters given in parentheses indicate typical possible relationships with other values. The values in italics are recognized as instrumental by both industry and university; the ones in bold are recognized as instrumental by industry, yet considered intrinsic by universities

Table 1 lists intrinsic values of industry and universities of technology as well as instrumental values that either directly support the intrinsic values, or support them via intermediated instrumental values. The number or letter in parentheses indicates the major correlation between values. For example in the middle column, job training, “i”, is instrumental for achieving good relations with future workers (d), which in turn helps industry to find good workers (b), which is instrumental for a business’s intrinsic task, namely to generate profit (A). Values between university and industry overlap: overlap in instrumental values is indicated in italics while highlighted in bold are values that industry recognizes as instrumental values, yet they are considered intrinsic values by the university. As an example, RWTH E considers advancement of knowledge in his field as an intrinsic value for both research and education purposes. In contrast, for the firms with which he collaborates, this is only instrumental in getting the CE label^10^ and improving their production process. This is the reason why collaboration for RWTH E stops if his industrial partners move beyond producing prototypes (see Value Conflict 2 above).

As was shown by the interviews with the university researchers, universities fulfill various goals. In particular, the interviewees referred to the three roles of universities corresponding to what they hold as intrinsically valuable: (1) generation of knowledge by doing research, (2) knowledge dissemination by teaching, and (3) contributing to applied research (e.g., Deiaco et al. 2012; Martin 2012). Moreover, at least recently, universities of technology commit themselves, for example via their mission statements, to contributing to solutions to societal problems, as exemplified by the “grand challenges” such as climate change and an increasingly aging population (Delft University of Technology 2019; RWTH Aachen 2013). With their mission statement, universities of technology underline that solving societal problems has intrinsic value for them. As a consequence, one could argue that universities of technology distinguish themselves from many other universities by stressing the great importance of knowledge dissemination in order to contribute to societal goals.

Whether values are intrinsic or instrumental often depends on the point of view. For example, training on the job done by students through internships in companies often contributes substantially to education at universities of technology. Therefore, job training within industry is intrinsically valuable for universities of technology. It is a key part of the education. At the same time, industry can profit from job training in two ways. First, student workers can directly contribute to the company’s profit, thereby being of intrinsic value. Second, internships can be a means to establish good relationships with potential employees with a good and relevant knowledge base. As this goal is subordinate to the company’s intrinsic value of making profit, it is of instrumental value. Another example is the development of prototypes which is of instrumental value to the university as it helps to disseminate knowledge. In contrast, it is of intrinsic value for industry because it may help to produce profit.

The values listed in Table 1 comprise the values mentioned in the interviews. For completeness, freedom of research as well as epistemic values for universities are also added. Both are obvious values that aim to secure the university’s aim of knowledge generation. While freedom of research is, in certain countries like Germany at least, an essential prerequisite for university research, epistemic values comprise a whole group of various values. These values may be empirical adequacy, simplicity of theoretical descriptions (for the sake of computability), comprehensiveness of the used model, precision (i.e., resolution), consistency with more fundamental theoretical descriptions such as the laws of thermodynamics, or even reducibility to more fundamental theories. In the wake of Thomas Kuhn’s landmark work *The Structure of Scientific Revolutions* (1970), various epistemic values can be seen as criteria for success in research. These values become of direct instrumental value in fulfilling the university’s goals when it comes to knowledge generation. For industry, some epistemic values are also of instrumental value as they help, for example, in developing a prototype. But other values, such as comprehensiveness or consistency, play a rather subordinate role for industry.

As discussed above, overlapping values lead to collaborations. For example, university as well as industry are interested in job training or prototype development. Nonetheless, serious conflicts arise from the different underlying intrinsic values. Some of these conflicts are depicted in Fig. 2. The most prominent cases of conflict arose due to conflicts between industry’s goal of making profit and the universities’ aim of knowledge dissemination, and between profit and education. Additional potential sources of conflict that were not addressed by the interviews are also indicated in the figure. One obvious conflict arises between the obligation of universities of technology to seek solutions to humanity’s grand challenges (societal goals), and industry’s primary goal of making a profit. In addition, various values within one organization may conflict. An example depicted in Fig. 2 is the potential conflict between the obligation of universities of technology to contribute to solving societal goals, and freedom of research.

Importantly, despite the aforementioned conflicts, universities and industry share many values, though these might not be of the same importance to both organizations. For example, while knowledge generation is intrinsic to universities, it is of instrumental value for industry. It is very valuable to acknowledge these positive relationships as it helps to make the point that university–business collaborations are not one-sided, or something that is merely enforced by policy makers, but rather of mutual benefit to both. Moreover, as detailed above, some of the aims of universities and industries can only be realized through close collaborations.

While the literature on university–business collaboration often focuses on research, our qualitative interviews showed that teaching and job training also require close collaboration. At the same time, job training may yield further conflicts. This tension is more pronounced for universities of technology as compared to universities in general. First of all, the education of engineers and architects requires hands-on training in their future jobs. This can only be achieved through close collaboration between industry and universities. Second, while some researchers at universities of technology do basic research, most focus on the third and fourth quadrant of the Stokes diagram (Fig. 1), i.e., use-inspired and pure applied research. This is exactly where industry-involvement is often inevitable.

The approach depicted in this paper of deriving values not from an underlying value theory, but rather from qualitative interview data, aligns with recent developments in applied ethics. These (in part) turn away from general debates on ethical theory in order to acknowledge that applied ethics is more than a straightforward application of general ethical principles to specific cases (Beauchamp 1984). Moreover, despite no moral theory being generally accepted, there seems to be a consensus on norms and values guiding tangible behavior (van de Poel and Royakkers 2011). The approach of Tom Beauchamp and James Childress (1979) to biomedical ethics in terms of general principles is an example at hand.

## Conclusions and Outlook

This paper identifies various value conflicts in university–industry collaborations. These results can be used for solving value conflicts by developing a framework along the lines of a recent approach within applied ethics of engineering, i.e., designing for values. A number of authors have developed approaches that aim to incorporate values into engineering or architectural design such as “values at play”, “value conscious design”, or “design for sustainability”. The most comprehensive account may be the “design for values” approach (van den Hoven 2005), although “value sensitive design” was developed by Batya Friedman and David G. Handry in the 1990s and they pioneered this work, initially focusing on ethical issues within information and communications technology (ICT) (Friedman 1997). The general idea in all these accounts has been applied—amongst others—to all kinds of engineering design (van de Poel 2009).^11^ All of these approaches try to offer a prospective account of ethical issues involved in the use of technology and thus aim at implementing various moral or social values during the design and development phase. Particularly the latter is important for detecting ethical issues, because in these early stages technologies and their social consequences are still malleable.

While designing for values has mainly been applied to the design of artifacts,^12^ it is also a framework for addressing the conflicts encountered in university–industry collaboration. It may prove useful both in devising specific research collaborations and even more, a suitable framework for providing more institutional guidance in the form of regulations. There are several noteworthy differences when applying a design for values approach to the “design” of university–industry collaborations, for example, in designing respective contracts between university and industry. Firstly, while in the design of material artifacts conflicts do not appear at the level of values, but rather at the level of norms or design requirements (van de Poel 2013), as indicated in the preceding sections, in university–industry collaboration one faces genuine value conflicts. Secondly, as within any university, knowledge generation is a value in itself; epistemic values also have importance here. While designing for values emphasizes ethical values, in order to deal appropriately with the conflicts in university–industry collaboration, one must also consider epistemic values. Here the question of how best to balance various epistemic values against societal goals is of particular relevance.

The values approach used to analyse university–industry collaborations facilitated examination of various forms of academic engagement in industrial activities, ranging from academic entrepreneurship through consultancy and publication of papers and patents (Perkmann et al. 2013). The range of conflicts that arise from university–industry collaborations are as diverse as these kinds of collaboration. With the help of detailed interviews and additional data we explored the complex landscape of potential conflicts in university–industry collaborations from the perspective of the underlying values. Interviews with engineers and architects at universities of technology illustrate the diverse conflicts of interest for individuals in these settings and inform the development of a theoretical concept.

As being aware of potential conflicts before they even emerge enables fruitful university–business collaboration, this study investigated conflicts in university–industry collaboration regarding values in order to highlight the underlying ethical conflicts. In particular, the value conflicts discussed consist of (1) students working in exchange for practical training, (2) public money being spent on applied research to make private profit, (3) the personal earnings of publicly funded researchers, (4) universities not being compensated for industrial use of their research, and (5) unfair competition resulting from the involvement of a publicly funded partner. When moving towards a values approach, the values discussed here were not derived from an underlying value theory. Rather, they were reconstructed from the interviews. The work in this paper can be seen as a first step towards a value-sensitive design approach to various kinds of university–industry collaborations. Concrete legal or organizational implications of this approach were not discussed.

University–industry collaborations are challenging for the individual researcher, but as our analysis showed the underlying problems are much more fundamental in the sense that they are not related only to the individual. The challenges relate to the aims of industry and the university, organizations of diverse people or departments. This makes it often even difficult to identify the overall values and goals of a particular firm or a particular university. The fundamental differences in values between university and industry result in difficult choices for applied scientists or engineers. Although university–industry collaborations are regulated at universities of technology, these generally leave unconsidered many problems that the individual academic faces. As the architect at RWTH Aachen University points out quite clearly, he considers the university–industry relationships an area where no rules exist that would fit his needs (RWTH A 01:10:02-9). In our view, the only way to solve conflicts is to clearly identify differences between university and industry with regard to the organizations’ values.

Three lines of research emerge from the results presented here. First, it would be helpful to develop solutions for value conflicts on a case-by-case basis. Second, it would be even more fruitful to develop general strategies for addressing value conflicts as this would make it much easier to initiate university–industry collaboration. At the same time, it is unclear whether further research on value conflicts will eventually lead to a set of rules that can guide researchers involved in university–industry collaborations. It might well be that value conflicts, and the circumstances under which they emerge, have such divergent characteristics that they have to be solved on a case-by-case basis. Still, an open, transparent and trustful discussion of value conflicts at the level of the individual, the university and the firm could help solve them in an efficient and fair way. As a third line of research, it would be worthwhile, though challenging, to find out whether and how the characteristics of the national innovation systems, the laws and codes of conduct that regulate and guide research in a given country, as well as the characteristics of the different disciplines, influence the kinds of value conflicts that emerge. All three lines of research discussed here would benefit from more interviews with researchers from different disciplines and countries as these interviews might reveal more detailed characteristics of value conflicts across disciplines and borders.

## Footnotes

1 For example, Katherine McComas (2012) reports that more than half of the nanotechnology researchers who responded to her survey depended on industry funding.

2 http://www.nwo.nl/onderzoek-en-resultaten/programmas/maatschappelijk+verantwoord+innoveren, in Dutch, last accessed 9 September 2019.

3 https://www.bmbf.de/de/der-spitzencluster-wettbewerb-537.html, in German, last accessed 9 September 2019.

4 http://www.raeng.org.uk/policy/dowling-review/the-dowling-review-of-business-university-research.

5 For a quick overview on the literature on engineering ethics, see the work of Pieter Vermaas and colleagues (2014).

6 One can either use probabilistic sampling when the sample is picked randomly, or theoretical sampling when the sample is picked according to criteria motivated by theory. In probabilistic sampling, the matched-pairs are usually formed by creating a sample randomly and then building a control group which matches the randomly chosen sample with regard to specific characteristics (e.g., age, seniority, rank). As an example, Arvanitis and Woerter (2009) analyzed collaborating and non-collaborating firms with the help of this method. A distinctively different route is theoretical sampling, where the sample is picked for theoretical reasons, i.e., particular cases are chosen that help to explore or even develop theory (Eisenhardt 1989; Eisenhardt and Graebner 2007).

7 Here the engineering sciences are distinguished from the sciences in general (the latter being comprised of both the natural and social sciences) and are more likely to lie in the upper left Stokes’ quadrant.

8 “The letters ‘CE’ appear on many products traded on the extended Single Market in the European Economic Area (EEA). They signify that products sold in the EEA have been assessed to meet high safety, health, and environmental protection requirements” http://ec.europa.eu/growth/single-market/ce-marking/, last accessed 19 July 2017.

9 RWTH A is referring to the research group within the department.

10 The CE label is a certification mark indicating conformity with health, safety, and environmental protection standards for any product sold within the European Economic Area.

11 Of course there are also limitations to value-sensitive approaches, see for example the work of Anders Albrechtslund (2007) and of Noëmi Manders-Huits (2011).

12 Exceptions are the integrated approach to a design for values in technical artifacts and institutions in the context of energy supply (Künneke et al. 2015).
